# Demand factor definition—A dimensionless parameter for Vertical Axis Wind Turbines

**DOI:** 10.1016/j.mex.2019.03.003

**Published:** 2019-03-20

**Authors:** Abdul Akbar M., Mustafa V.

**Affiliations:** a Department of Civil Engineering, National Institute of Technology, NIT Campus (P.O), Calicut, Kerala, 673 601, India; b Department of Civil Engineering, Madanapalle Institute of Technology & Science, Madanapalle, 517325, India

**Keywords:** Demand factor approach, Wind energy, Vertical axis wind turbine, Optimization, Streamtube, Demand factor, Lift, Drag, Power, Analytical, DMST

## Abstract

The use of dimensionless numbers like Reynolds Number, Froude Number and Webber Number has historically simplified the process of comparison of phenomena irrespective of their scales and in their classification into different categories. This paper deals with the derivational aspects of a dimensionless parameter named “Demand Factor” for optimization of Vertical Axis Wind Turbine (VAWT).

• The input parameters considered in this derivation are power, wind velocity, the aspect ratio of the turbine, density of air and viscosity of air and the output parameters are length of the blade, number of blades, chord length, aerofoil shape, radius of the turbine and angular velocity at rated speed.
• Four rounds of variable definition trials are carried out through the arrangement of the input parameters on the numerator and denominator positions. With the filtering out of unsuitable combinations at different stages of elimination, out of 32 combinations the expression that holds the potential to represent demand factor was identified. The process of carrying out single point optimization based on Demand factor expression is discussed along with the steps involved in numerically calculating output parameters.
• The expression of Demand factor developed provides a different perspective on the process of design and optimization of VAWTs.

## Graphical abstract

**Figure:**
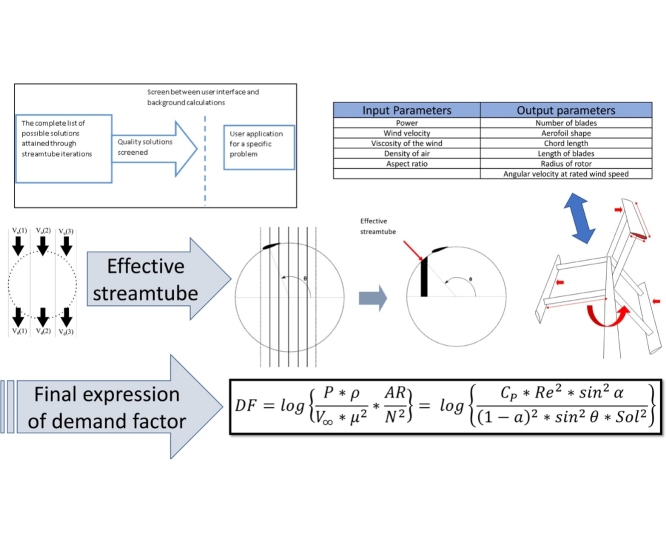

**Specifications Table**

**Table:** 

**Subject Area:**	• Energy • Engineering
**More specific subject area:**	Wind Energy
**Method name:**	Demand Factor approach
**Name and reference of original method:**	Original methods is as per reference [13] M. Abdul Akbar and V. Mustafa, A new approach for optimization of Vertical Axis Wind Turbines, J. Wind. Eng. Ind. Aerodyn. 153, 2016, 34–45.
**Resource availability:**	NA

## Method details

### Introduction

The field of energy needs newer methods [1,2] in its battle to sustain its role as the chief driver of economy. In this context, enhanced research is required in the field of renewable energy especially wind energy which is currently the fastest growing source. Although Vertical Axis Wind Turbines (VAWTs) are inherently less efficient than Horizontal Axis Wind Turbines, their usage has increased in the past two decades. Numerous methods have been proposed and used for solving the flow physics of VAWTs. Out of all the methods, solutions based on streamtube theories stand out. The easiness of the approach makes up for its inaccuracy compared to more accurate approaches like Computation Fluid Dynamics. The first reference to the classical streamtube theories was by Templin [3] in the year 1974 with the introduction of the single streamtube theory as an ‘Aerodynamic Performance Theory’ for VAWTs. The effects of aerodynamic stall and curvature of the blade on performance was incorporated. In the same year, Robert et al. [4] in a report with the primary focus on Horizontal Axis Wind Turbines (HAWTs) introduced the concept of using multiple independent streamtubes for analysis. The streamtube models gained further momentum when James on behalf of Sandia National Laboratories wrote a report in 1975 [5] comparing the single and multiple streamtube models with experimental results. The report confirmed better prediction capabilities of multiple streamtube models when compared to single streamtube models for small rotors. Lapin [6] briefly introduced the concept like DMST of having two actuator disks in tandem in 1975 when he developed it to study the economic feasibility of a large rotor.

**Fig. 1:**
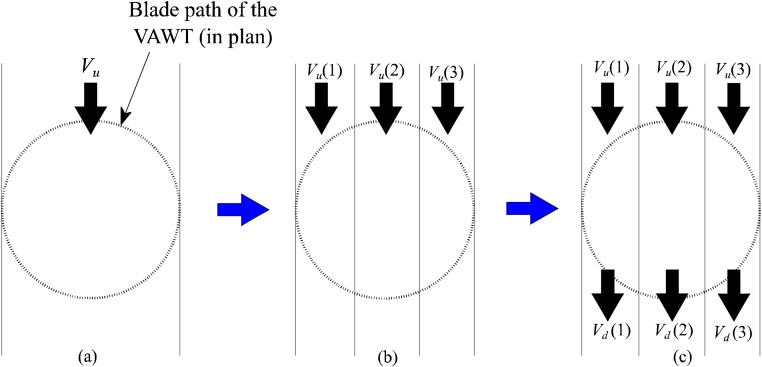
Evolution of streamtube theory: (a) Single streamtube theory, (b) Multiple streamtube theory, (c) Double multiple streamtube theory.

Classical streamtube theories have gone through immense transformation over the years. Out of these, three phases are the most prominent and is often referred to in discussions about the history of streamtube theories. They are, the transformation from ‘Single streamtube theory’ to ‘Multiple streamtube theory’ to ‘Double multiple streamtube theory’ (DMST). Fig. 1 shows a simple representation of these phases.

Concurrent with the development of the theory was its usage for optimization of VAWTs. An aerodynamic optimization method for straight bladed Darrieus VAWTs based on the streamtube theories was proposed in 1983 [7]. DMST was used to develop a mathematical optimization model to enhance the power coefficient of vertical axis turbine for tidal current conversion by adjusting the blades deflection angle [8]. Optimizing studies on the rotor of the VAWT were carried out with variables as radius of the rotor, number of the blades, chord length and blade height [9]. Use of genetic algorithm [10] and consideration of generality of shape of VAWT and wind direction [11] in optimization have demonstrated the versatility of streamtube theories. Design guidelines for optimization of annual energy yield of H-Darrieus wind turbines have been proposed [12] by the usage of streamtube theories.

This paper deals with the derivational aspects of a non-dimensional parameter named “Demand Factor” for optimization of VAWT. The paper discusses a modified version of the Demand Factor definitions first introduced in [13] along with the modified approach of carrying out the optimization process. The study is a continuation of research that has been pointing in the direction of single point optimization whereby the lift and drag coefficients were attempted to be modelled using equations by regression analysis [14].

## Aerodynamics of VAWT based on DMST

**Fig. 2:**
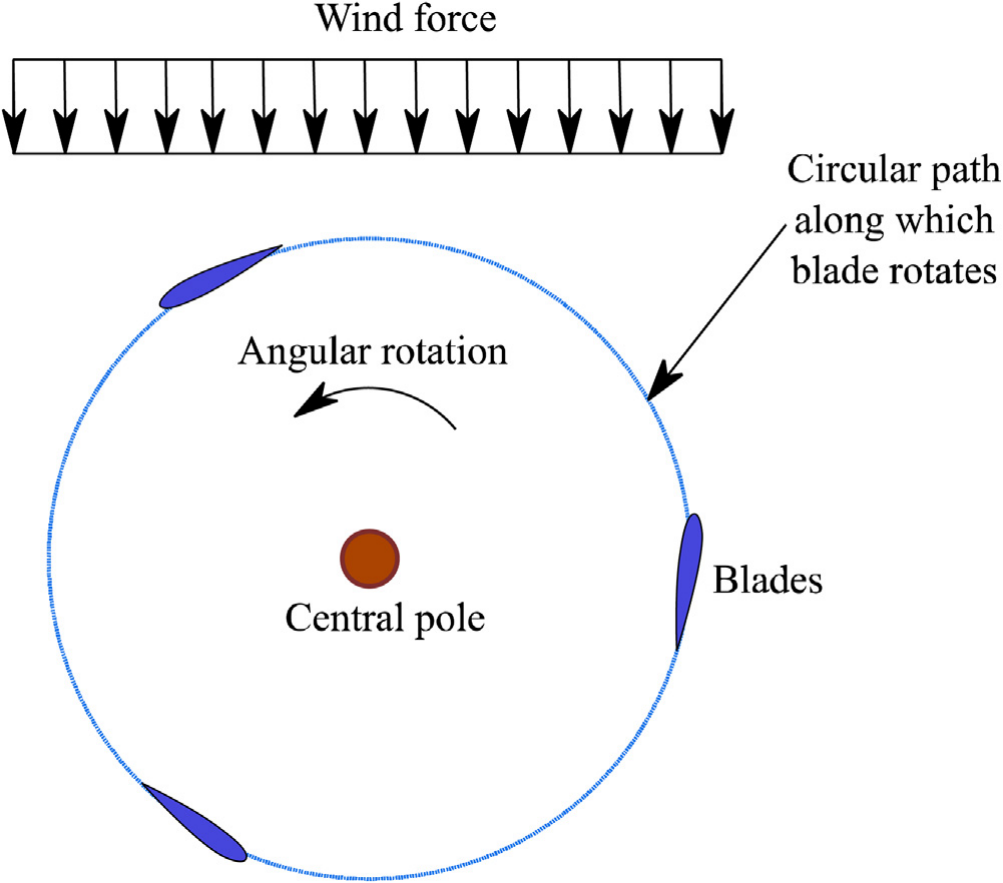
Schematic plan of VAWT depicting aerodynamicss.

**Fig. 3:**
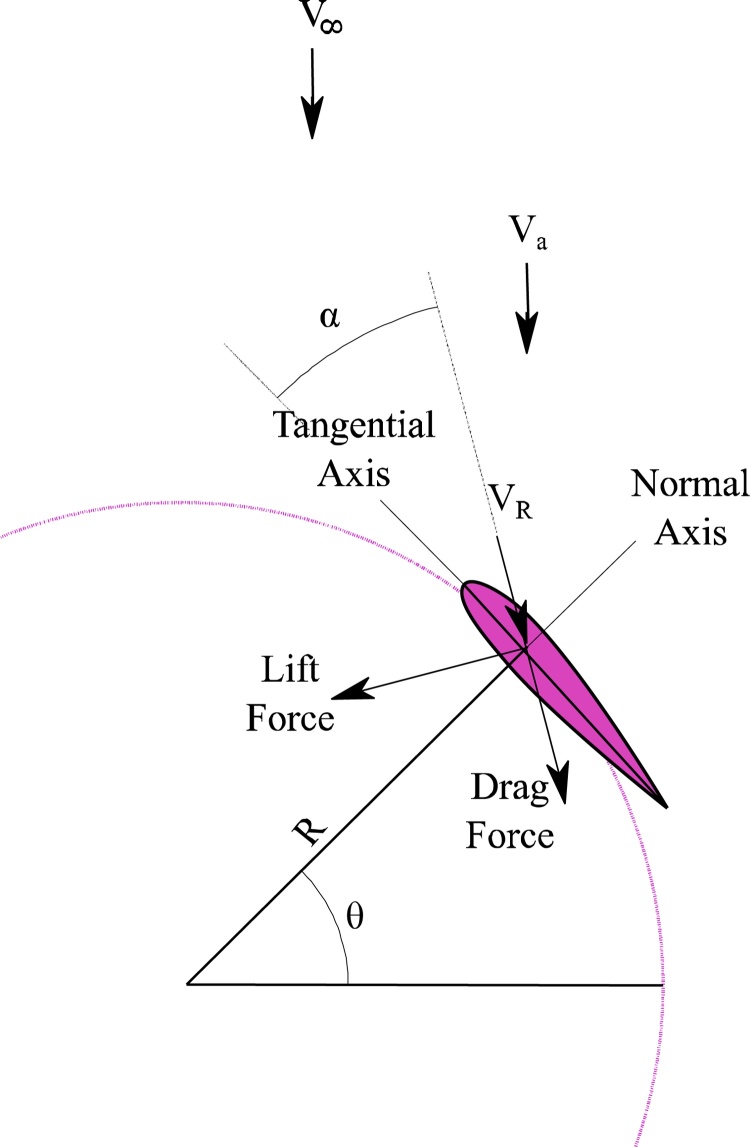
VAWT blade section subjected to forces.

The established formulas relating to aerodynamics of VAWTs for necessary for demand factor derivation are discussed in this section. Consider the plan view of a 3-bladed VAWT as shown in Fig. 2 and a close-up view of a blade as shown in Fig. 3. During operation, the VAWT is simultaneously subjected to wind force from an arbitrary direction and its own angular rotation about the central pole. Relative velocity due to the net effect of two forces acting together is given by Eq. (1).

(1) $$V_{R}^{2}={\left(V_{a}*\sin\theta \right)}^{2}+{\left(V_{a}*\cos\theta +\omega *R\right)}^{2}$$

Where, *V_R_* is the relative velocity, *θ* is the azimuthal position of the blade section, *V_a_* is the axial flow velocity that is lower than the external wind velocity *V_∞_* owing to the combined effect of the forces. *ω* is the angular velocity of the VAWT which varies instantly with the change in wind velocity, and *R* is the radius of the rotor. The term ω*R in Eq. (1) accounts for the variation of relative velocity due to rotation of VAWT.

Taking square root on both sides and dividing it by *V_∞_*, Eq. (1) becomes

(2) VRV∞=1-asinθ2+1-acosθ+λ2

Where, *λ* denoted as tip speed ratio is given by,

(3) $$\lambda =\;\omega *R/V_{\infty }$$

“*a”* referred to as axial induction factor is defined as,

(4) a= 1-VaV∞

The relative velocity owing to the combined action of forces can be obtained using Eq. (2). The direction of the resultant could be found out similarly from the basic geometry and is given as:

(5) $$\tan\alpha =(1-a)\sin\theta /\left\{\left(1-a\right)\cos\theta +\lambda \right\}$$

The equation can also be re-written using trigonometric function sine and Eq. (5) becomes,

(6) sinα=(1-a)sinθ/VRV∞

During the operation of VAWT, a resultant wind velocity whose magnitude is given by Eq. (2) hits the blade section along its direction given by Eq. (6). As with all other fields that involve a fluid-solid interaction, only a part of the force will be effective in the direction of the force whereas the remaining part will be effective in pushing the solid in a direction perpendicular to the line of action of force. The proportion of these forces (in parallel and perpendicular directions) depends upon the property of the aerofoil corresponding to the Reynolds number of operation and angle at which the wind hits the aerofoil. In aerodynamics, these force coefficients are defined as lift (*C_L_*) and drag coefficients (*C_D_*).

(7) $$C_{L}=f(Re,\;\alpha ,\;Shape)$$

(8) $$C_{D}=f(Re,\;\alpha ,\;Shape)$$

The values of *C_L_* and *C_D_* cannot be determined exactly as they are functions of shape (in this case aerofoil) which can have wide spectrum of forms with no general rule to define geometry. Here, Reynolds number (*Re*) is given as,

(9) $$Re=\rho *V_{R}*C/\mu$$

Where, *ρ* is the density of air, *V_R_* is the relative velocity calculated from Eq. (2), *C* is the chord length of the aerofoil and *μ* is the viscosity of air.

With reference to Fig. 3, it is evident that for every position of the aerofoil, there is a corresponding normal and tangential component direction that is instantaneous. Resolving the lift and drag coefficients in the direction of normal and tangential components, we get,

(10) $$C_{n}=C_{L}*\cos\alpha +\;C_{D}*\sin\alpha$$

(11) $$C_{t}=C_{L}*\sin\alpha -C_{D}*\cos\alpha$$

The instantaneous thrust forces developed due to the combined action of the forces can be written as,

(12) $$T_{i}=0.5*\rho *V_{R}^{2}*L*C*(C_{t}*\cos\theta -C_{n}*\sin\theta )$$

Where *L* is the height of the blade. The component of force which is responsible for the angular rotation of blade referred to as instantaneous torque, *Q_i_* is calculated as,

(13) $$Q_{i}=0.5*\rho *V_{R}^{2}*L*C*C_{t}*R$$

According to the theoretical formulations of DMST, the VAWT is divided in plan into multiple adjacent but aerodynamically independent streamtubes and computations are carried out through each of those independent streamtubes. The final solution is arrived at by the summation of results obtained through them. The number of streamtubes used should be sufficient to ensure a converged solution. Most of the works of literature identify 36 streamtubes as a converged value.

**Fig. 4:**
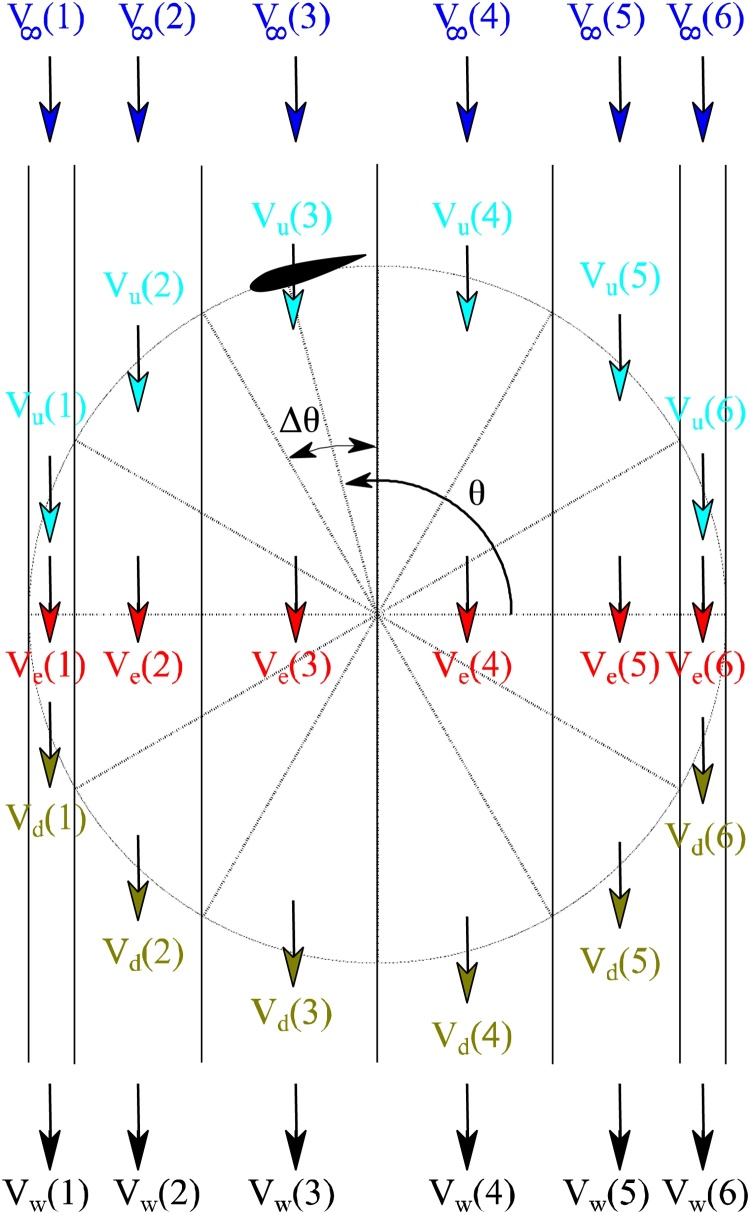
The division of VAWT into number of aerodynamically independent streamtubes.

As per DMST formulations, the wind is required to pass through each streamtube twice before reaching the other end. The calculations are carried out separately for the upstream and downstream halves of the rotor. The streamtube divided computational geometry is depicted in Fig. 4.

If Δ*θ* is the angular dispersion of the streamtubes and *N* is the number of blades, then the time-averaged thrust force, *T_a_* is given as,

(14) $$T_{a}=N*T_{i}*2*\Delta \theta /\pi$$

The average thrust coefficient, *C_T_* can be written as,

(15) $$C_{T}=T_{a}/\left(0.5*\rho *V_{\infty }^{2}*L*R*\Delta \theta *\sin\theta \right)$$

After substituting the value of *T_a_* from Eq. (14), we get

(16) CT=NCπR*VRV∞2Ctcosθsinθ-Cn

Betz thrust coefficient for an ideal wind turbine is equated against Eq. (16) to obtain the value of induction factor *a* for each streamtube. This equality for induction factor values less than 0.4 is (Eq. (17)).

(17) 4a1-a=NCπR*VRV∞2Ctcosθsinθ-Cn

The resulting equation is of the complex quadratic type that can be solved only through a trial and error procedure. The torque coefficient $\left(C_{Q}\right)$ and power coefficient $(C_{P}$) are given by,

(18) $$C_{Q}=\left(\frac{NC}{2R}\right)*\sum_{i=1}^{2m}\frac{\left[{\left(\frac{V_{R}}{V_{\infty }}\right)}^{2}*C_{t}\right]}{2*N_{s}}$$

Where, the summation is carried out upto twice the number of streamtubes (*2 m*), considering both upstream and downstream directions.

(19) $$C_{P}=C_{Q}*\lambda$$

Total power of the VAWT is calculated as,

(20) $$P=C_{P}*\rho *R*L*{V_{\infty }}^{3}$$

It is evident from Eqs. (16) and Eq. (18) that the variables: number of blades (*N*), chord length (*C*) and Radius (*R*) appear in a format in relation to each other. This formation is denoted by the term solidity (*Sol*) which is defined by Eq. (21).

(21) Sol= N*C/R

The ratio of radius to the height of the VAWT is defined as aspect ratio (*AR*).

(22) $$AR=\;\frac{R}{L}$$

## Prelude to development of demand factor

**Fig. 5:**
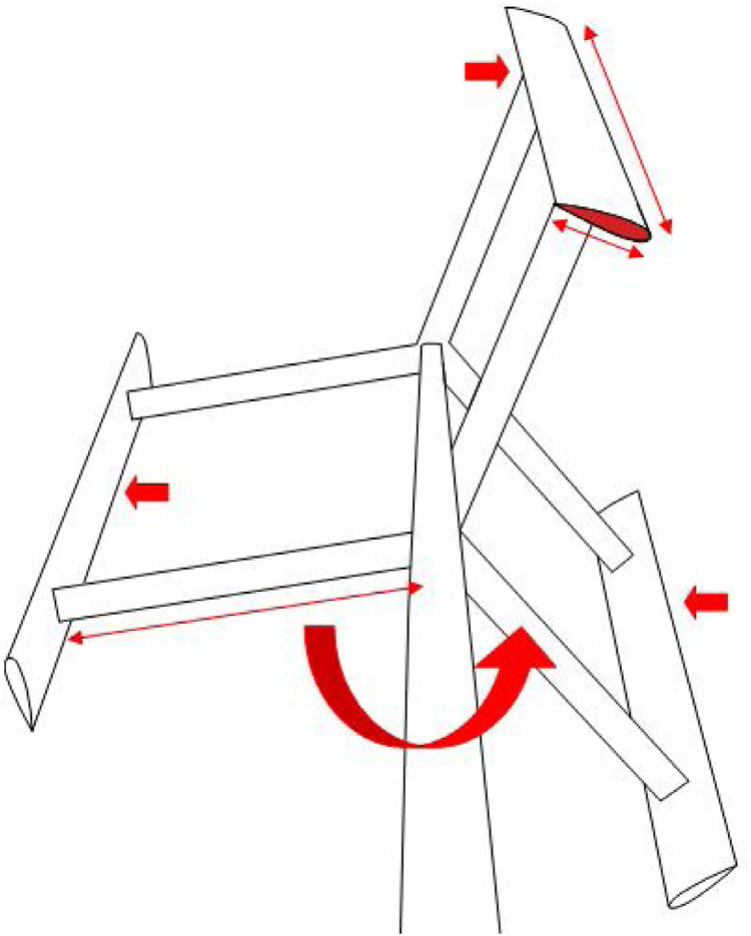
Parameters in the design of VAWT.

The input parameters in the design of VAWT are rated power, rated wind speed, aspect ratio, density of air & viscosity of air. The latter two are dependent on the location and altitude of VAWT installation. For the simplest VAWT, which is straight bladed and has no pitch variations, at least six parameters are required to design it completely. They are: length of the blade, radius of the rotor, number of blades, aerofoil shape, chord length of the blade and target angular rotation at rated wind speed. The output parameters of VAWT are shown in Fig. 5.

**Fig. 6:**
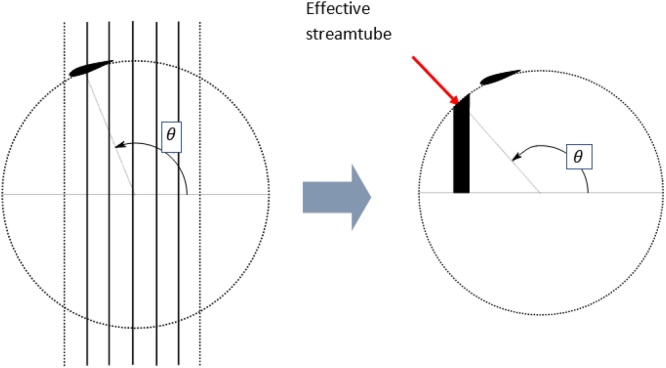
Concept of effective streamtube.

One of the hurdles of the streamtube theories (other than single streamtube theory) in the development of a single point optimization program is that the performance (power coefficient) of the entire VAWT could be calculated only after calculating the performances of all the individual streamtubes (as evident from Eqs. (18) and (19)). To circumvent this issue and to provide a single point reference for VAWTs, the concept of effective streamtube is introduced. An effective streamtube is defined as the streamtube corresponding to the azimuthal position that gives the power coefficient closest to overall power coefficient of the VAWT calculated with a specified number of streamtubes. In other words, the entire VAWT with certain number of streamtubes is replaced by a single streamtube that can represent the VAWT. The concept is represented graphically through Fig. 6.

The concept of isolating a streamtube has been carried out earlier by Conaill [15] through the definition of effective lift to drag ratio based on the calculation of average torque per cycle. However, although the earlier definition is also about representing the entire VAWT by the properties of a particular streamtube, a new definition as discussed above was necessary due to the following reasons:

1) The benchmarking based on power coefficient helps to maintain the entire calculations in non-dimensional regime.
2) The coefficient of power is a direct quantification of the efficiency of the VAWT.
3) Lift to drag ratio has a larger range of values and has values in the negative regime making it difficult to handle.
4) The earlier definition does not specify a minimum number of streamtubes for its definition to be valid.

The variables involved in DMST analysis can be classified into four categories. They are listed in Table 1. The first column contains all the known parameters based on which an optimized design needs to be obtained. The second column presents all the unknown parameters for which the design must be carried out in the most optimized manner. The fourth column contains the control parameters. Based on changing any of the control parameters, an entirely new solution is arrived at with its intrinsic properties of effective streamtube as shown in the third column of Table 1. Upon changing the values of *Re*, *Sol* and TSR for different aerofoil shapes, an infinite number of solutions with their corresponding properties of effective streamtube is obtained. The challenge is to get the most optimized solution from the pool of solutions that satisfies the input parameters.

**Table 1:** Classification of variable for development of optimization algorithm.

Known parameters	Unknowns	Possible solution (effective streamtube)	Control parameters
*P*	*N*	Induction factor (*a*)	Reynolds number (*Re*)
*V_∞_*	Aerofoil shape	Power coefficient (*Cp*)	Tip speed ratio (*λ*)
*μ*	*C*	Relative velocity (*V_R_*)	Solidity (*Sol*)
*ρ*	*L*	Azimuthal position (*θ*)	Aerofoil shape
*AR*	*R*	Angle of attack (*α*)	
	*ω*	Correction factor	

## Derivation of demand factor

Based on the classification of variables into four categories as shown in Table 1 and using the equations of aerodynamics (Eqs. (1), (2), (3), (4), (5), (6), (7), (8), (9), (10), (11), (12), (13), (14), (15), (16), (17), (18), (19), (20), (21), (22)), different expressions of the known and unknown parameters were obtained after re-arrangements within themselves in a certain relation to each other as shown in Table 2. The unmarked columns indicate the respective mathematical operators.

**Table 2:** Rearrangement of equations for use in the development of Demand Factor.

Variables		Dependent variables					Remarks
		Known		Unknown		Solution	
P	*=*	$\rho *{V_{\infty }}^{3}$	*	R*L	*	$C_{P}$	Eq. (20)
$V_{\infty }$	*=*			ω*R	*/*	λ	Eq. (3)
μ	*=*	ρ	*	C	*	$V_{R}/Re$	Eq. (9)
ρ	*=*	ρ					Dummy Eqn.
AR	*=*			R/L			Eq. (22)

All the equations of Table 2 are obtained after re-arranging them in terms of known variables that control the design. The following rules were adopted in developing the expression for demand factor (DF):

1) Expression has to be non-dimensional
2) It has to contain all the input variables of Table 1 on one side of the equation
3) It has to contain properties of the possible solution on the other side of the equation
4) It may not contain any of the output variables of Table 1 as they are unknowns until the completion of design

Combining five variables into a single term produces a total of 2^5^ or 32 combinations (Table 3) including the option that each variable can be combined separately as part of either numerator or denominator. In the table, "N" stands for the numerator and "D" stands for the denominator, indicating the position occupied by the variable in the expression. RHS stands for the right-hand side of the expression obtained by using formulations of Table 2.

**Table 3:** Combinations tried for defining the DF.

	*P-V_∞_-ρ-μ-AR*	RHS		*P-V_∞_-ρ-μ-AR*	RHS
1	N-N-N-N-N	$C_{P}*\rho^{2}*R^{2}*V_{\infty }^{4}*\mu$	17	D-N-N-N-N	$\mu /(C_{P}*L^{2}*V_{\infty }^{2})$
2	N-N-N-N-D	$C_{P}*\rho^{2}*L^{2}*V_{\infty }^{4}*\mu$	18	D-N-N-N-D	$\mu /(C_{P}*R^{2}*V_{\infty }^{2})$
3	N-N-N-D-N	$C_{P}*\rho^{2}*R^{2}*V_{\infty }^{4}/\mu$	19	D-N-N-D-N	$1/(C_{P}*L^{2}*V_{\infty }^{2}*\mu )$
4	N-N-N-D-D	$C_{P}*\rho^{2}*L^{2}*V_{\infty }^{4}/\mu$	20	D-N-N-D-D	$1/(C_{P}*R^{2}*V_{\infty }^{2}*\mu )$
5	N-N-D-N-N	$C_{P}*R^{2}*V_{\infty }^{4}*\mu$	21	D-N-D-N-N	$\mu /(C_{P}*\rho^{2}*L^{2}*V_{\infty }^{2})$
6	N-N-D-N-D	$C_{P}*L^{2}*V_{\infty }^{4}*\mu$	22	D-N-D-N-D	$\mu /(C_{P}*\rho^{2}*R^{2}*V_{\infty }^{2})$
7	N-N-D-D-N	$C_{P}*R^{2}*V_{\infty }^{4}/\mu$	23	D-N-D-D-N	$1/(C_{P}*\rho^{2}*L^{2}*V_{\infty }^{2}*\mu )$
8	N-N-D-D-D	$C_{P}*L^{2}*V_{\infty }^{4}/\mu$	24	D-N-D-D-D	$1/(C_{P}*\rho^{2}*R^{2}*V_{\infty }^{2}*\mu )$
9	N-D-N-N-N	$C_{P}*\rho^{2}*R^{2}*V_{\infty }^{2}*\mu$	25	D-D-N-N-N	$\mu /(C_{P}*L^{2}*V_{\infty }^{4})$
10	N-D-N-N-D	$C_{P}*\rho^{2}*L^{2}*V_{\infty }^{2}*\mu$	26	D-D-N-N-D	$\mu /(C_{P}*R^{2}*V_{\infty }^{4})$
11	N-D-N-D-N	$C_{P}*\rho^{2}*R^{2}*V_{\infty }^{2}/\mu$	27	D-D-N-D-N	$1/(C_{P}*L^{2}*V_{\infty }^{4}*\mu )$
12	N-D-N-D-D	$C_{P}*\rho^{2}*L^{2}*V_{\infty }^{2}/\mu$	28	D-D-N-D-D	$1/(C_{P}*R^{2}*V_{\infty }^{4}*\mu )$
13	N-D-D-N-N	$C_{P}*R^{2}*\mu *V_{\infty }^{2}$	29	D-D-D-N-N	$\mu /(C_{P}*\rho^{2}*L^{2}*V_{\infty }^{4})$
14	N-D-D-N-D	$C_{P}*L^{2}*\mu *V_{\infty }^{2}$	30	D-D-D-N-D	$\mu /(C_{P}*\rho^{2}*R^{2}*V_{\infty }^{4})$
15	N-D-D-D-N	$C_{P}*R^{2}*V_{\infty }^{2}/\mu$	31	D-D-D-D-N	$1/(\mu *C_{P}*\rho^{2}*L^{2}*V_{\infty }^{4})$
16	N-D-D-D-D	$C_{P}*L^{2}*V_{\infty }^{2}/\mu$	32	D-D-D-D-D	$1/(\mu *C_{P}*\rho^{2}*R^{2}*V_{\infty }^{4})$

From the linear combinations performed and shown in Table 3, it is evident that no combinations prove to simplify the expression for DF. However, an aspect of the combinations was noted that was helpful for the second round of variable definition study. It is the appearance of variables, power, density of air and viscosity of air on the same side (numerator or denominator) of the fraction that would make elimination of density of air from RHS impossible as it would lead to a variable that is raised to the cubic power of density of air. Thus, to overcome this situation, only those combinations were considered which did not have all these three combinations on either the numerator or the denominator. From Table 3, the combinations 1, 2, 9, 10, 23, 24, 31 and 32 possess the above situation and were eliminated from the second round of combination development trials.

Further, a complete elimination of density of air from RHS would be possible only if one of the variables amongst power, viscosity of air and density of air were squared and the variables are placed in such a manner that the squared variable lies on the opposite side of the fraction (numerator or denominator) compared to other two variables. It was decided to square viscosity of air, and hence to ensure a complete elimination of density of air from RHS, only those combinations that had the viscosity of air variable on opposite side of the fraction (numerator or denominator) compared to power and density of air were selected for the second round. Therefore, the combinations of 5, 6, 7, 8, 13, 14, 15, 16, 17, 18, 19, 20, 25, 26, 27 and 28 were eliminated from going into the second cycle. The remaining combinations (3, 4, 11, 12, 21, 22, 29 and 30) in a simplified form and re-numbered sequentially for the second round of arriving at the definition of DF are shown in Table 4.

**Table 4:** Second round of combination definitions.

	*P-V_∞_-ρ-μ-AR*	RHS	RHS simplified by Eq. (9)
1	N-N-N-D^2^-N	$C_{P}*\rho^{2}*R^{2}*V_{\infty }^{4}/\mu^{2}$	$C_{P}*{Re}^{2}*R^{2}*V_{\infty }^{4}/\left(V_{R}^{2}*C^{2}\right)$
2	N-N-N-D^2^-D	$C_{P}*\rho^{2}*L^{2}*V_{\infty }^{4}/\mu^{2}$	$C_{P}*{Re}^{2}*L^{2}*V_{\infty }^{4}/\left(V_{R}^{2}*C^{2}\right)$
3	N-D-N-D^2^-N	$C_{P}*\rho^{2}*R^{2}*V_{\infty }^{2}/\mu^{2}$	$C_{P}*{Re}^{2}*R^{2}*V_{\infty }^{2}/\left(V_{R}^{2}*C^{2}\right)$
4	N-D-N-D^2^-D	$C_{P}*\rho^{2}*L^{2}*V_{\infty }^{2}/\mu^{2}$	$C_{P}*{Re}^{2}*L^{2}*V_{\infty }^{2}/\left(V_{R}^{2}*C^{2}\right)$
5	D-N-D-N^2^-N	$\mu^{2}/(C_{P}*\rho^{2}*L^{2}*V_{\infty }^{2})$	$\left(V_{R}^{2}*C^{2}\right)/C_{P}*{Re}^{2}*L^{2}*V_{\infty }^{2}$
6	D-N-D-N^2^-D	$\mu^{2}/(C_{P}*\rho^{2}*R^{2}*V_{\infty }^{2})$	$\left(V_{R}^{2}*C^{2}\right)/C_{P}*{Re}^{2}*R^{2}*V_{\infty }^{2}$
7	D-D-D-N^2^-N	$\mu^{2}/(C_{P}*\rho^{2}*L^{2}*V_{\infty }^{4})$	$\left(V_{R}^{2}*C^{2}\right)/C_{P}*{Re}^{2}*L^{2}*V_{\infty }^{4}$
8	D-D-D-N^2^-D	$\mu^{2}/(C_{P}*\rho^{2}*R^{2}*V_{\infty }^{4})$	$\left(V_{R}^{2}*C^{2}\right)/C_{P}*{Re}^{2}*R^{2}*V_{\infty }^{4}$

From Table 4, it is evident that the variables, density of air and viscosity of air were completely removed. However, unknowns such as the radius, height and the chord length of the blade are still present. The variables of chord length and radius appearing on the opposite sides of the fraction (numerator or denominator) could be modified as per the equation for solidity given by Eq. (21). Similar simplifications are not available with the ratio of the height and the chord length of the blade. Therefore, the combinations that contain the ratio of the latter were eliminated and hence the combinations 2, 4, 5 and 7, of Table 4, were chosen out from going into the third round. The remaining four combinations are shown in Table 5.

**Table 5:** Third round of combination definitions.

	*P-V_∞_-ρ-μ-AR*	RHS	RHS simplified by Eq. (21)
1	N-N-N-D^2^-N	$C_{P}*{Re}^{2}*R^{2}*V_{\infty }^{4}/\left(V_{R}^{2}*C^{2}\right)$	$C_{P}*{Re}^{2}*N^{2}*V_{\infty }^{2}/\left\{{\left(V_{R}/V_{\infty }\right)}^{2}*{Sol}^{2}\right\}$
2	N-D-N-D^2^-N	$C_{P}*{Re}^{2}*R^{2}*V_{\infty }^{2}/\left(V_{R}^{2}*C^{2}\right)$	$C_{P}*{Re}^{2}*N^{2}/\left\{{\left(V_{R}/V_{\infty }\right)}^{2}*{Sol}^{2}\right\}$
3	D-N-D-N^2^-D	$\left(V_{R}^{2}*C^{2}\right)/C_{P}*{Re}^{2}*R^{2}*V_{\infty }^{2}$	$\left\{{\left(V_{R}/V_{\infty }\right)}^{2}*{Sol}^{2}\right\}/(C_{P}*{Re}^{2}*N^{2})$
4	D-D-D-N^2^-D	$\left(V_{R}^{2}*C^{2}\right)/C_{P}*{Re}^{2}*R^{2}*V_{\infty }^{4}$	$\left\{{\left(V_{R}/V_{\infty }\right)}^{2}*{Sol}^{2}\right\}/(C_{P}*{Re}^{2}*N^{2}*V_{\infty }^{2})$

From Table 5, it is seen that first combination is same as the second combination except that there is an additional term ${\;V}_{\infty }^{2}$. A similar observation was noted for the fourth combination with regards to the third combination. Taking advantage of the above observation and with an objective of defining non-dimensional form for the DF, the 1st and 4th combinations maybe eliminated, ultimately ending up with the two combinations listed in Table 6.

**Table 6:** Fourth round of combination definitions.

	*P-V_∞_-ρ-μ-AR*	RHS	RHS simplified by Eq. (6)
1	N-D-N-D^2^-N	$C_{P}*{Re}^{2}*N^{2}/\left\{{\left(V_{R}/V_{\infty }\right)}^{2}*Sol\right\}$	$C_{P}*{Re}^{2}*N^{2}*{\text{sin}}^{2}\alpha /\left\{{\left(1-a\right)}^{2}*{\text{sin}}^{2}\theta *{Sol}^{2}\right\}$
2	D-N-D-N^2^-D	$\left\{{\left(V_{R}/V_{\infty }\right)}^{2}*Sol\right\}/(C_{P}*{Re}^{2}*N^{2})$	$\left\{{\left(1-a\right)}^{2}*{\text{sin}}^{2}\theta *{Sol}^{2}\right\}/C_{P}*{Re}^{2}*N^{2}*{\text{sin}}^{2}\alpha$

The definitions of Table 6 are reciprocals of each other. Out of these, the first combination was chosen over the second as having power in the numerator would provide a better physical feel. The first combination can be written as:

(23) $$\frac{P\text{*}AR\text{*}\rho }{V_{\text{∞}}\text{*}\mu^{2}}=\;\frac{C_{P}\text{*}{Re}^{2}\text{*}N^{2}\text{*}\sin^{2}\alpha }{\left\{{\left(1-a\right)}^{2}\text{*}\sin^{2}\theta \text{*}{Sol}^{2}\right\}}$$

Re-arranging, we get,

(24) $$\left(\frac{P\text{*}\rho }{V_{\text{∞}}\text{*}\mu^{2}}\right)\text{*}\left(\frac{AR}{N^{2}}\right)=\;\frac{C_{P}\text{*}{Re}^{2}\text{*}\sin^{2}\alpha }{\left\{{\left(1-a\right)}^{2}\text{*}\sin^{2}\theta \text{*}{Sol}^{2}\right\}}$$

From Eq. (24), it can be seen that the left hand side (LHS) contains all the known parameters of design that are listed in Table 1. Besides, there is also the term for the number of blades which is an unknown until the completion of the design. However, unlike other unknowns, the number of blades is easier to work with as they can take only discrete values of 2, 3, 4, 5, etc.

The RHS of Eq. (24) corresponds to the properties of the effective streamtube that qualify to be a solution. RHS does not contain any of the input or output parameters that are listed in Table 1. Thus, Eq. (24) represents a unique way of relating the input variables of VAWT design to the intrinsic variables of the possible solution in a way that is not seen presented so far by works of literature. The LHS and RHS of the equation are non-dimensional. The presence of square of Reynolds number in Eq. (24) indicates a significant order of magnitude for the numbers. Hence, considering natural logarithm of both sides, the formulation for demand factor (DF) can be completed as given in Eq. (25).

(25) $$DF=\log\left\{\frac{P\text{*}\rho }{V_{\text{∞}}\text{*}\mu^{2}}\text{*}\frac{AR}{N^{2}}\right\}=\;\log\left\{\frac{C_{P}\text{*}{Re}^{2}\text{*}\sin^{2}\alpha }{{\left(1-a\right)}^{2}\text{*}\sin^{2}\theta \text{*}{Sol}^{2}}\right\}$$

The developed formulation of DF satisfies all the four objectives set forth except the appearance of the number of blades (*N*) in the formulation that is an unknown until completion of the design.

## Expression of DF for computations

With the definition of demand factor (DF), a platform for comparison of input variables and intrinsic variables of the possible solution is obtained. The LHS of the expression for DF given by Eq. (25) contains the input parameters of design and is termed as input demand factor (IDF). The RHS of the equation contains the internal properties of the possible solution and is termed as the aerodynamic demand factor (ADF). Furthermore, the VAWT manufacturer cannot insist on a fixed value of aspect ratio as that will make the design very rigid. However, an acceptable range of aspect ratio is specified, and any design that falls in that range is taken as a solution to the problem. With regards to the number of blades, there will be a minimum and maximum number of VAWT blades from considerations of erection feasibility and easiness of manufacture. As long as the solution obtained is within this permissible range of the number of blades, the solution is deemed acceptable. Therefore, IDF can have a range of values depending upon the values of aspect ratio and number of blades. The extreme limits are identified as the minimum and maximum values of IDF. The four definitions are shown through Eqs. (26), (27), (28), (29).

(26) $$IDF=\log\left\{\frac{P\text{*}\rho }{V_{\text{∞}}\text{*}\mu^{2}}\text{*}\frac{AR}{N^{2}}\right\}$$

(27) $$ADF=\;\log\left\{\frac{C_{P}\text{*}{Re}^{2}\text{*}\sin^{2}\alpha }{{\left(1-a\right)}^{2}\text{*}\sin^{2}\theta \text{*}{Sol}^{2}}\right\}$$

(28) $${IDF}_{min}=\log\left\{\frac{P\text{*}\rho }{V_{\text{∞}}\text{*}\mu^{2}}\text{*}{\left(\frac{AR}{N^{2}}\right)}_{min}\right\}$$

(29) $${IDF}_{max}=\log\left\{\frac{P\text{*}\rho }{V_{\text{∞}}\text{*}\mu^{2}}\text{*}{\left(\frac{AR}{N^{2}}\right)}_{max}\right\}$$

**Fig. 7:**
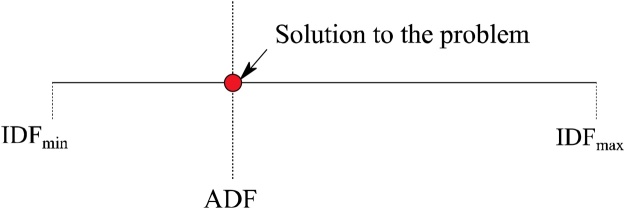
An effective streamtube solution for the given problem.

For a particular “effective streamtube” to be a feasible solution to the optimization problem, the ADF value for it should lie within the limits of minimum and maximum values of IDF calculated. This situation is demonstrated graphically in Fig. 7.

## Selection of optimum solution

There could be numerous effective streamtubes that are eligible to become the solution to the given problem. As an optimization problem, the goal is to find the most efficient solution. Two measures are employed to ensure that the resulting solution is the optimum.

**Fig. 8:**
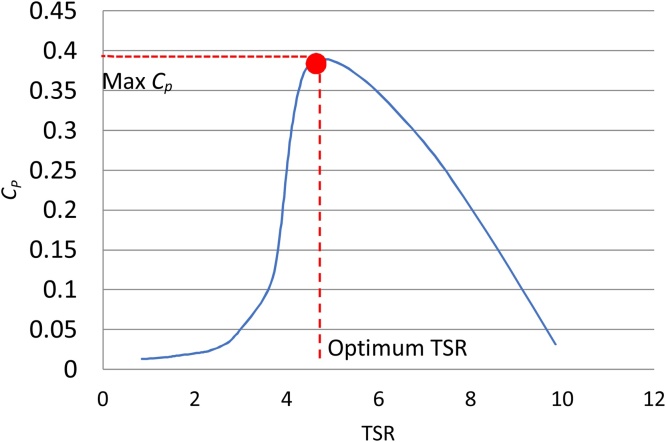
TSR versus *C_p_* showing the Optimum TSR.
2) **Seeking solution from descending order of power coefficients -** The solution set would contain properties of different effective streamtubes corresponding to optimum TSRs of various combinations. These different cases are arranged in descending order of power coefficients. Once the IDF_min_ and IDF_max_ for a problem are calculated, the range of values is compared with the ADF values calculated for the list of effective streamtubes starting from the first one. Arrangement in descending order will ensure that the optimal solution is selected as the topmost entry satisfying the ADF requirement.

1) **Choosing the optimum TSR** – For an analysis, there is a value of Tip Speed Ratio (TSR) that would yield the maximum power coefficient (Fig. 8). Thus, one of the ways of ensuring an optimum solution is to extract only those effective streamtubes that correspond to the optimum TSR.

## Multiple solutions

**Fig. 9:**
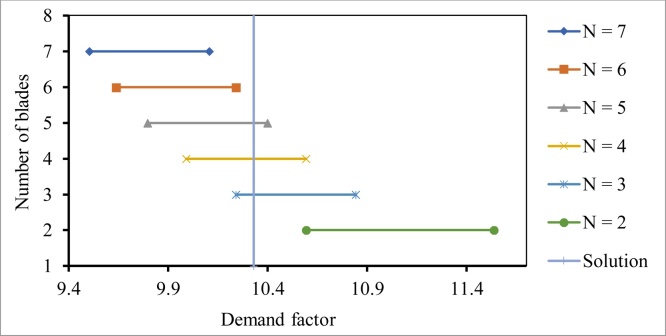
Demonstration of case involving multiple solutions.

In certain situations, multiple solutions are obtained that provide the same amount of efficiency. The situation occurs in cases where the ADF falls within the range of values where different sets of number of blades can offer the same ***C_p_*** within the maximum range of aspect ratio possible. This situation is represented graphically in Fig. 9.

Back calculations for all the three cases would yield different sets of solution (different length, radius, chord length, etc.), all of them giving the same value of power coefficient. Among these solutions, the one that gives the value of angular velocity at rated speed that is closest to the comfort zone of the manufacturer is selected as the final solution.

## No valid solution for analysis

**Fig. 10:**
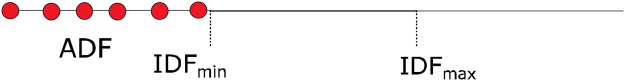
IDF range greater than all the ADF’s.
2) The IDF range is within the limits of ADF values of the effective streamtube set but still misses the solution (Fig. 11). This case indicates that the resolution of control parameters (fourth column of Table 1) is too coarse to provide a solution.

In certain situations, there are no effective streamtubes that have ADF value between the range of IDF_min_ and IDF_max_ calculated. This could happen in one of the following scenarios:

1) The range of IDF is greater than all the ADF values of the effective streamtube set (Fig. 10). It means that with the current limitation of the number of blades and aspect ratio, it is not physically possible to attain the specified power.

**Fig. 11:**
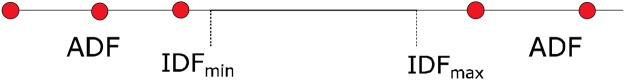
IDF ranges between ADF’s but misses the solution.
3) The range of IDF is lesser than all the ADF values of the effective streamtube set (Fig. 12). This case is very unlikely, but it means that the demanded power output from the VAWT is too low.

**Fig. 12:**
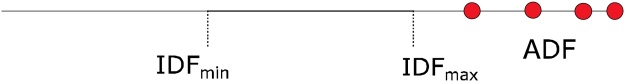
IDF range lesser than all the ADF’s.

## Conclusion

A variable called “demand factor” is developed for the optimization of VAWTs. This variable was developed considering four different criteria. First, it should be non-dimensional, secondly, it should contain all the input variables on one side of the expression. Then, it should contain the intrinsic variables on the other side of the equation and finally, it should not contain any output variables as they are unknowns until the completion of the design. The left hand side of Demand Factor expression contains the input variables and is identified as a range that is termed as input demand factor (IDF). The expression on the RHS contains the various intrinsic variables of design that is labelled as the aerodynamic demand factor (ADF).

For optimization process, a large set of effective streamtube solutions are generated by varying the values of control parameters. Only the effective streamtubes corresponding to the value of optimum TSR for a case of analysis are selected from the above set. These effective streamtubes are then arranged in the decreasing order of their power coefficients. The first effective streamtube that has an ADF value that falls within the range of IDF values is taken as the solution to the problem. Thus, through this process, the definition of Demand Factor enables a single point optimization wherein five input variables are uniquely connected with six output variables of VAWT.
